# Role of consumer ethnocentrism on purchase intention toward foreign products: Evidence from data of Vietnamese consumers with Chinese products

**DOI:** 10.1016/j.heliyon.2023.e13069

**Published:** 2023-01-18

**Authors:** Ngoc Ha Nguyen, Trung Kien Dao, Thanh Thuy Duong, Tuan Thanh Nguyen, Van Ky Nguyen, Thi Lanh Dao

**Affiliations:** Faculty of Business and Economics, Phenikaa University, Nguyen Trac, Yen Nghia, Ha Dong, Ha Noi, Viet Nam

**Keywords:** Chinese-origin products, Country image, Consumer ethnocentrism, Product image, Purchase intention

## Abstract

This study aims to determine the influence of consumer ethnocentrism and general country image on Vietnamese consumers' perception of product origin and purchase intention toward Chinese goods. The research model is developed based on consumer ethnocentrism and country image perception theory. Analysis results by structural equation modeling from 448 consumers in three regions (North, Central, South) showed that consumer ethnocentrism harms the country’s image perception and purchase intention of Vietnamese consumers toward Chinese imported products. However, consumer ethnocentrism does not significantly affect the product country image perception. On the contrary, both the general country image and product country image positively impact Vietnamese consumers' intentions to buy Chinese imported goods. Finally, the study also points out some limitations and implications for later studies and marketers dealing with domestic products in Vietnam.

## 1 Introduction

With a long history of commercial and cultural exchanges, the bilateral economic cooperation relationship between Vietnam and China is one of the most solid and profound relations between China and countries. However, this is also a sensitive relationship due to many political and historical factors. According to Xia [1], more than half of Vietnamese respondents feel China has the most significant influence in Asia. Vietnam has become China's largest trading partner in ASEAN and is now one of China's six most prominent trading partners globally [2]. Meanwhile, China remains Vietnam's largest trading partner, the market's significant commodities provider, and the country's second-largest export market (after the US) [3]. Chinese goods are becoming increasingly popular among Vietnamese consumers, with large-scale imports including computers, machinery, materials for garment and footwear industries, and smartphones [4].

Despite being ideologically similar (communism) and heavily influenced by China's politics, the anti-China sentiment of Vietnamese consumers is quite clear. Due to the complicated historical relationship, there are often conflicts between Vietnam and China through wars in history, most recently in 1979, actions such as forcibly occupying the Paracel Islands (Hoang Sa in Vietnamese) in 1974 or the recent Haiyang Shiyou 981 oil rig violating Vietnam's exclusive economic zone [5]. These actions have led to increased economic nationalism, which involves favoring one's own country over others [6], and anti-China sentiment in Vietnam that may hinder Chinese imports. In addition, due to complicated historical and political relations, Chinese goods are often questioned about product quality or simply the anti-China sentiment of Vietnamese consumers. According to a survey by Xia [1], only about 25% of Vietnamese consumers surveyed believe China's influence is positive.

The country image indicates consumers' perceptions, which reflect consumers' beliefs in a country's economy, culture, people, and products [7]. Country image partly contributes to a product’s total image, which helps buyers evaluate the price, quality, durability, and risk when purchasing products from a particular country [8]. According to previous studies, consumers tend to have positive or negative evaluations of products created in a foreign country; thus, origin biases have been discovered in developed and developing countries [9]. In general, the latter's products are thought to be uncertain and of poorer quality than the former's. In the present day, Chinese products, or the tag "Made in China" are seen everywhere in the world. Contrary to the somewhat negative impression of consumers about Chinese goods in the past, now, as the world's largest exporter and the second largest economy in the world after the US, China has developed a variety of goods industries with the expectation of affirming its position in the hearts of consumers. As the market grows over time, the quality and its brand reputation should also follow.

When it comes to product purchases, Vietnamese people place a high priority on the country of origin. This is especially true for people in their 30s with high wages [10]. The wave of protectionism, or economic nationalism, is being harnessed by domestic businesses worldwide against the entry of foreign goods. Movements that call for the use of domestically produced goods have attracted the attention of many researchers. The concept of consumer ethnocentrism is mentioned in the studies to reflect the tendency of customers to choose products or services based on their origin [11]. Consumer ethnocentrism supposes that buying goods that originated from other countries is disloyal and can harm the domestic economy [12].

Vietnamese consumers significantly prefer products made in more developed countries [13]. Furthermore, according to Ref. [14], the desire for foreign items is prevalent, with 77% of Vietnamese consumers surveyed indicating a preference for overseas products over domestic ones. As a result, imported products have a significant impact on Vietnamese consumer behavior regarding quality and prices.

In Vietnam, although China is a significant trading partner and also a significant exporter to the Vietnamese market [3], the "anti-Chinese" sentiment or suspicion against Chinese goods is quite common [15]. In response to the pressure from Chinese goods, many Vietnamese manufacturers have launched the slogan "Vietnamese people use Vietnamese goods," "using Vietnamese goods is patriotic" [16] to attract customers based on national pride, which is the premise of consumer ethnocentrism. Accordingly, consumer ethnocentrism refers to consumers' beliefs about the ethical suitability of purchasing foreign-made products [17]. Highly ethnocentric consumers think buying imported products is wrong because it does not benefit domestic production and is unpatriotic.

While there have been many studies on the consumer ethnocentrism of different customer groups with imported goods in the world [18]. However, such studies have not been done much in Vietnam because Vietnamese consumers are regarded as a customer group with a tendency to prefer imported goods. Moreover, the special relationship between Vietnam and China has been formed throughout history, and Chinese goods are gradually becoming the most direct competitors for Vietnamese enterprises. Meanwhile, studies on the consumer ethnocentrism of Vietnamese consumers, national image perception, and the tendency to buy Chinese goods have not been carried out. Therefore, research on consumer ethnocentrism and intention to buy products of Chinese origin has both scientific and practical significance for Vietnamese enterprises. Therefore, in this study, we aim to clarify the influence of consumer ethnocentrism and general country image on Vietnamese consumers' perception of product origin and purchase intention toward Chinese goods.

## 2 Literature review

### 2.1 Purchase intention of foreign products

The concept of purchase intention has its origin in the concept of “intention” in behavioral theories such as the theory of planned behavior (TPB) [19]. Accordingly, intention is seen as the individual's willingness or capacity to perform a particular behavior [19]. Likewise, for foreign products that are imported, the purchase intention of foreign products reflects a consumer's willingness to purchase and use these products [20]. In marketing, purchase intention is used as a concept for the long-term prediction of buying behavior as it relates to the willingness of potential customers to buy [21].

### 2.2 Consumer ethnocentrism

The concept of ethnocentrism is a term in sociology which refers to a feeling of one's group superiority, considering one's group preference [20]. Research in consumption was first proposed by Shimp & Sharma [22] for US consumers and was defined as "the beliefs held by American consumers about the appropriateness, indeed morality, of purchasing foreign-made products" (p.280). Consumers with high consumer ethnocentrism will think that using imported products is wrong and unethical because it is detrimental to the economy and unpatriotic. Such consumers may perceive the consumption of imported products as unethical, which could lead to the loss of jobs for domestic workers [20]. Therefore, consumers belonging to a highly ethnocentric group will prefer to buy and use domestic products and have less favorable attitudes toward foreign products and brands [20,23,24]. This leads to the judgment of countries and their products based not on an objective treatment of available information but on stereotyped images of these countries. For example, a study by Luis-Alberto et al. [25] presents a neuroimaging experiment to examine Spanish consumers’ attitudes towards products made in Spain, the USA, and China. Results reveal that domestic products are more likely to elicit feelings of satisfaction among consumers and are preferred over imported ones. However, ethnocentrism is unlikely to have an effect in the absence of domestic or domestically produced products [26,27].

Consumer ethnocentrism is used as a tool in international marketing activities to promote economic nationalism in refusing to buy foreign products and preferring domestic products [26,28,29]. People with high consumer ethnocentrism tend to avoid buying imported products, some have an aversion to buying products from specific countries and express negative perceptions about those countries, although they consider it acceptable to buy products from other countries [26,28]. Therefore, it can be speculated that highly ethnocentric people may negatively perceive the country's image or product image, not based on objective judgments but on stereotypes and perceptions of that country [26].

### 2.3 General country image and product country image

According to Roth & Diamantopoulos [30] general country image, or in short country image, is seen as a general concept made up of generalized images created by the degree of economic and political maturity, historical events and relationships, culture and traditions, and the degree of technological skill and industrialization. All of these elements pertain to cognitive beliefs about a specific nation [30].

Some other scholars consider country image in its role as origins of products [30], or in different words, country of origin. For example, Li et al. [31] define country image as “consumers' images of different countries and of products made in these countries”. However, according to Ref. [32], the term product country image presents more accurately the underlying meaning of country image in the sense of product origins. The country of origin effects and the product country image effects could be used interchangeably [33].

Country of origin is seen as a signal of product quality [34,35] to influence perceived risk, as well as purchase intention [7,36]. Country of origin was found to significantly influence consumers' opinion of products and the likelihood of purchasing these products [37,38]. Studies have provided substantial evidence supporting that country of origin creates country image and plays an important role in creating awareness of that country's products and brands [7,39]. Because country image is viewed as descriptive, inferential, and informative beliefs and perceptions about a particular country [40]. Country of origin studies show that country of origin biases exist in all products in general as well as specific products [41,42] in both developed and less developed countries [43], in both end consumers and institutional customers [44,45].

The general image of the country is considered to have a direct influence on consumers’ product confidence and an indirect influence on the ability to accept a product [26]. Research by Laroche et al. [7] shows that regardless of the level of familiarity with products manufactured in a particular country, consumers use both country image and beliefs in the product image to shape buying attitudes. Therefore, it can be considered that country of origin creates the perception of the country's image through the values and prejudices of consumers about that country and affects the perception of the product or brand image from the country.

### 2.4 Research model and hypotheses

**Fig. 1:**
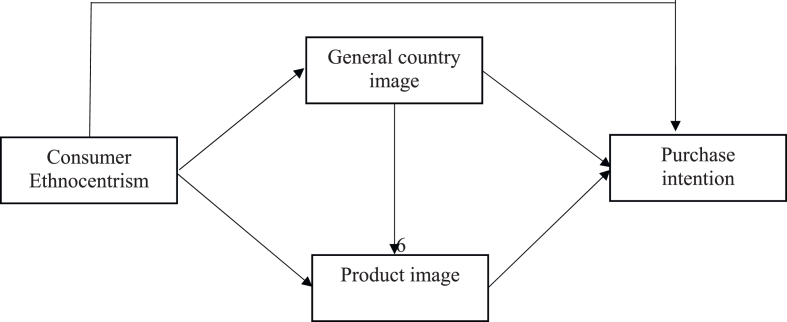
Research model proposed.

As discussed above, consumer ethnocentrism is considered to influence perceptions of country image or beliefs about products from a specific country in a stereotyped pattern that is not necessarily based on objective information. The country-of-origin theory explains that perceptions of the country image lead to prejudices against products or brands from a particular country and affect the receptivity of products [26]. Therefore, in this study, we develop a model of the relationship between consumer ethnocentrism and country perception (general country image and product image) with the purchase intention of products imported from China by Vietnamese consumers. The research model is presented in Fig. 1; the mechanism of influence of constructs in the research hypothesis from the model is detailed in the hypothesis explanation.

### 2.5 Hypotheses

#### 2.5.1 Consumer ethnocentrism

As discussed above, ethnocentric customers consider the act of purchasing foreign goods as non-ethnic, which negatively affects their own country's economies and causes unemployment, while customers with weak consumer ethnocentrism evaluate imported products only based on products’ values and characteristics, regardless of their origins [46,47]. Therefore, consumer ethnocentrism may have an effect on the country's image perception or national status of imported goods. However, there have not been many studies examining this relationship except for [48]. Even in the study of De Nisco et al. [26], they only found evidence of the relationship between the Spanish customer group and goods imported from Germany but did not find this effect among the Italian customer group. This is the research gap that we want to examine further in the context of Vietnam.

There are several research findings show that the degree of Vietnamese consumer ethnocentrism serves as a significant driver of consumption in Vietnam, positively impacts the intention to purchase local Vietnamese products [49,50]. These findings implicate that for Vietnamese people, national pride in consumption is important to them. Moreover, Vietnam and China have a sensitive relationship due to historical issues and complicated political relations, which leads to a commonly observed notion that Vietnamese consumers have strong anti-China sentiments. A survey by Singapore's ASEAN Studies Center illustrates that more than 90% of Vietnamese people feel worried about the influence of China's economy, 97.7% worried about China's political influence in Vietnam [51]. This phenomenon could be a boost for consumer ethnocentrism of Vietnamese to be even higher, especially towards Chinese imported products, and could have effect on Vietnamese perception of China's country image. Therefore, in this study, we argue that there is an influence of consumer ethnocentrism on the perception of national image.

H1 Consumer ethnocentrism of the Vietnamese has a negative impact on the country's image of China.

Consumer ethnocentrism also affects the way consumers perceive imported goods [52,53]. Highly ethnocentric customers may view buying products from outside of the group as immoral and promote products "in-group" [22,26,54]. This leads to the consequence that highly ethnocentric consumers may express a negative view toward imported goods as belonging to the “out-group” regardless of other information about it. This leads to biased judgments of consumers regarding imported goods from particular countries [26]. Highly ethnocentric consumers may judge the products of some particular countries based on their preconceived notions, the stereotypes they ascribe to those countries. Therefore, it can be speculated that highly ethnocentric customers will have a less positive attitude towards imported products. Therefore, this study hypothesized.

H2 Consumer ethnocentrism of the Vietnamese has a negative impact on a product's image of China

Consumer ethnocentrism is regarded as a tool to promote sales of domestic products and limit the sale of imported products [23]. Customers who are highly ethnocentric are often reported to have more positive reviews of domestic products than those without nationalist tendencies [20,23]. Studies show that there is a significant link between consumer ethnocentrism and willingness to buy imported products [20,23,55]. Previous studies have shown that consumer ethnocentrism has a negative impact on consumers' purchase intention of foreign products [20,23,26]. This leads businesses to use nationalist incitement as a subtle way to protect domestic producers from the competition of imported products [20]. Therefore, we continue to propose the hypothesis.

H3 Consumer ethnocentrism of the Vietnamese has a negative impact on purchase intention toward Chinese products

#### 2.5.2 Country image and product image

Country image is associated with the general representation of a specific country and its people, including perceptions of the country’s political, economic, and technological development stages, its social and political systems, stereotypes and reputation, and in particular, the quality of the products made in that given country [56,57]. Country image is considered one of the important influencing factors in evaluating products from different origins. Product image refers to consumers' meaningful perceptions or attitudes of a particular product [58]. When specific information about a product is not available, or customers are not familiar with the brand, they often rely on their prior perceptions of the product image of certain given countries. They associate each country and its products with certain attributes (for example, its economic or technological development level), which influences how they perceive products coming from these countries. Hence, customers' evaluation of a certain product, such as its quality, symbolic value, design, durability, or credibility, is influenced by the product's national identity stemming from its original country [59]. A positive country image can produce a strong emotional connection to certain brands and products and enhance a product's attractiveness; thus, customers are more likely to have greater confidence in the quality of products [60]. The relationship between country image and product image has been verified through various studies [48,61,62]. We hypothesize that country image has significant effects on product perception of Chinese products.

H4 Country image has a positive impact on the Chinese product image

A positive relationship between country image and customers’ purchase intention has been established by many researchers [62,63]. It refers to the buyer's decision to purchase a product based on their expectation of the country where it was made. Consumers' positive impression of a brand's country of origin results in a positive attitude toward the brand itself and the product preference [64]. Moreover, Pappu et al. [65] indicated that country image affects their loyalty towards the brands originated from that country. Positive country image can lead to brand popularity and eventually to consumer brand loyalty, so whenever faced with the choice of one among many products of the same kind, customers may use the country image as an attribute to eliminate the alternatives and purchase their favorite brand. When consumers do not know much about the product, their perceived country image could be substituted for product image to evaluate product quality, leading to purchase intention [62]. On the contrary, buyers who have a hostile impression or attitude toward a particular country may not decide to purchase a brand originated from that country, regardless of its specific qualifications [66]. When customers encounter an unusual foreign brand product, the lack of familiarity with the given product may lead to unfavorable assumptions about the brand's quality, and they may consider not buying it. It is also argued that country image has a more significant effect on purchase intention when customers are not familiar with its products than when they are [67]. Therefore, it is hypothesized that country image has a positive impact on purchase intention of Chinese products.

H5 Country image has a positive impact on purchase intention of Chinese products

Product image refers to consumers' general perceptions through the image or qualification of a particular country's products [58], or the total beliefs consumers have regarding products of a given country [56]. While country image stimulates customers’ interest in purchasing when they are unfamiliar with its products, product image seems to have a greater direct impact [62]. When buyers are acquainted with a clear product image from a foreign country, they will directly assess the product quality based on their knowledge about the product information, such as high workmanship, great design, strong durability to make choice decisions. This leads to the following hypothesis.

H6 Product image has a positive impact on purchase intention of Chinese products

## 3 Methodology

### 3.1 Sample

Our potential surveyed subjects are identified as Vietnamese consumers who have experience using Chinese goods within the last year. The survey was carried out in all three areas: the North, the Central, and the South of Vietnam. With a sampling error of no more than 5%, we calculated the minimum sample size needed for the study to be 384 [68]. Therefore, the expected sample size to be investigated was 400 to guarantee the reliability of the quantitative analysis. The survey was carried out online through the Google Form, sent to shopping groups on the internet, and through the authors' snowball method. The study also was approved by the scientific ethics committee at Phenikaa University. The survey participants were explained about the purpose of the study and confirmed to participate completely voluntarily according to an informed consent form before conducting the survey. The survey was conducted for two months, from June to July 2021. As a result, 448 valid questionnaires were obtained and being used for the study.

Despite using convenience sampling method, our survey results have certain similarities with the characteristics of typical Vietnamese consumers. In our survey sample, the low-income group who earn under 5 million/month accounts for about 50% of the population, Deloitte's survey data shows that about 40% of employees are in the low-income group [69]. In addition, in Deloitte's survey, low-income, price-sensitive people tend to prefer Chinese products, while high-income people prefer Japanese, Korean and Western products [69]. The characteristics of surveyed customers are described in Table 1.

**Table 1:** The profile of respondents.

		Frequency	Percent
Gender	Men	163	36.4
	Women	285	63.6
Occupation	Government officer	64	13%
	Student	237	49%
	Self-employed	41	8%
	Office staff	94	19%
	Other	52	11%
Income	<5 mil (VND)	227	50.7
	5–10 mil (VND)	70	15.6
	10–15 mil (VND)	49	10.9
	15–20 mil (VND)	37	8.3
	20–25 mil (VND)	23	5.1
	>25 mil (VND)	42	9.4
Experience (in using Chinese products)	<1 year	79	17.6
	1–3 years	127	28.3
	3–5 years	77	17.2
	5–7 years	32	7.1
	7–10 years	14	3.1
	>10 years	119	26.6

### 3.2 Measure

The constructs in the model were adapted from literature review and previous studies [18,48,62,[70], [71], [72]]. Specifically, the consumer ethnocentrism construct was evaluated by six items based on Klein et al., and Narang [70,71]. The country image was measured by five items referenced from De Nisco et al. [26], and Wang et al. [73]. The product image construct was evaluated by seven items based on De Nisco et al., Roth and Diamantoulos [26,30]. The purchase intention construct was referenced from studies of Narang; and Diamantopoulos et al. [20,74] with six items on the scale. The items in each construct were translated from English to Vietnamese and used the back-translation method to ensure that they did not change the original meaning of the questions in the questionnaire. The draft questionnaire was corrected through a discussion by a focus group with five experts in the marketing field. Then the questionnaire was conducted a pilot test with 40 potential respondents to adjust the presence of each question in the questionnaire before the official survey. The items are described in Table 2. The Likert scale was used to measure for each item with 1 – totally disagree and 5 – totally agree.

**Table 2:** Results of reliability and convergent validity of each construct.

Constructs/Items	Statement	Factor loadings
***Consumer Ethnocentrism (****Adapted from* Refs. [20,54]*):* Mean = 3.438, SD = 0.877, α = 0.805, CR = 0.838, AVE = 0.567		
ETH1	I think only products that cannot be produced or are not available in our country should be imported.	Remove
ETH2	I always think that local products should always be the first priority of choice when shopping.	0.656
ETH3	As a Vietnamese, one should prioritize buying Vietnamese goods whenever having the opportunity.	0.796
ETH4	Although I may suffer a minor loss of benefits, I will always support and choose to buy domestic products when possible.	0.798
ETH5	Vietnamese people should support and buy Vietnamese products; otherwise, we will enrich only foreign countries.	0.752
ETH6	Vietnamese people should not buy imported goods because it will hurt the domestic economy and increase unemployment.	Remove
***General country image (****Adapted from* Refs. [26,73]): Mean = 3.11, SD = 0.832, α = 0.765, CR = 0.790, AVE = 0.652		
GCI1	I think the Chinese people are trustworthy	Remove
GCI2	I think China is now a high-tech country	Remove
GCI3	I think the Chinese people today have a high level of literacy.	0.827
GCI4	I think the Chinese people now have a high standard of living.	0.788
GCI5	I think China is a friendly country to Vietnam.	Remove
***Product country image (****Adapted from* Refs. [26,30]*):* Mean = 2.847, SD = 0.682, α = 0.837, CR = 0.858, AVE = 0.548		
PCI1	I think products imported from China are highly innovative these days	Remove
PCI2	I think products imported from China usually have nice designs.	Remove
PCI3	I think products imported from China are from reputable brands.	0.717
PCI4	I think products imported from China have a high level of sophistication or require high skills.	0.710
PCI5	I think products imported from China use modern technology.	0.662
PCI6	I think products imported from China are high quality.	0.850
PCI7	I think products imported from China are durable.	0.750
***Purchase intention (****Adapted from* Refs. [20,74]): Mean = 3.109, SD = 0.759, α = 0.885, CR = 0.878, AVE = 0.548		
INT1	I support product importation from China	0.717
INT2	When possible, I will buy goods imported from China.	0.812
INT3	I will buy imported goods from China if they are available.	0.823
INT4	I will buy products imported from China if I have a need for specific products.	0.655
INT5	I will tell my family and friends about imported Chinese products' good and positive characteristics.	0.704
INT6	I will recommend goods imported from China to others.	0.715

### 3.3 Data analysis method

The study used the multivariate analysis method to analyze surveyed data and test hypotheses in the model. The confirmatory factor analysis (CFA) was used to test model fit and reliability, and validity of each construct [75]. The criteria of CFA include Chi-square/df is less than 3; CFI, TLI, IFI are all greater than 0.9, and RMSEA is less than 0.08 [[75], [76], [77], [78]] showing the model fit with the actual data [77]. To evaluate the convergent validity of each construct, we use the criteria the factor loading of items is greater than 0.6 [78]. In order to test discriminant validity, we used the comparison between the square root of average variance extracted (AVE) with any correlation coefficients of constructs [75]. If the square root of AVEs is greater than the correlation among constructs showing, the constructs reach the discriminant validity [79]. We use the criteria to test the reliability: Cronbach's Alpha coefficients, composite reliability coefficients are larger than 0.7, and AVE is larger than 0.5 [79]. Structural equation modeling was used to test hypotheses with statistical significance at a 0.05 level. In addition, we also use the direct, indirect, and total effect coefficients to evaluate the impact of mediator variables on dependent variables in the model.

### 3.4 Common method and non-response bias

Research that uses the survey method to collect data can lead to the influence of common method bias on research conclusions [80]. Therefore, we have used several methods to reduce the influence of common method bias while designing the study. As suggested by Ref. [81], we used anonymous respondents, carefully designed questionnaires, and used statements that reflect both the positive and negative aspects of the questionnaire. Collected data using Harman's test with a unique factor in factor analysis indicates that total variance explained is less than 50% (30.178%). This shows that common method bias is not an issue in this study.

Non-response phenomenon can also be a problem of research when essential respondents are overlooked. To test whether non-response affects the research results or not, we use the *t*-test to compare the values of the variables at the early stage of the research and the late stage of the research. As suggested by Ref. [82] we split the data in a ratio of 70:30 by early and late periods. The analysis results did not find any difference between the early and late groups in the mean values of the items (p-value >0.05). That shows that the non-response phenomenon did not affect the results.

## 4 Results

### 4.1 Reliability and validity

The analysis result indicated that the model achieves fit with the actual data: Chi-square/df = 2.750 was less than 3; CFI = 0.947; TLI = 0.936; IFI = 0.947 were all greater than 0.5; and RMSEA = 0.063 was less than 0.08. The factor loading of each item in the CFA analysis was greater than 0.6, presenting that the constructs in the model achieved convergent validity. The Cronbach's Alpha and composite reliability coefficients were larger than 0.7, and the average variance extracted was larger than 0.5 (Table 2). This finding showed the constructs in the model are reliable.

To test the discriminant validity of each construct in the model, we used the criteria the square root of AVEs compared with any correlation coefficients among constructs. The findings indicated that the square root of constructs was larger than the correlation coefficients of constructs showing that the constructs in the research model reached discriminant validity (Table 3).

**Table 3:** Discriminant validity of each construct in the model.

Constructs	(1)	(2)	(3)	(4)
Consumer ethnocentrism (1)	**0.753**			
General country image (2)	−0.146	**0.807**		
Product country image (3)	0.053	0.570	**0.740**	
Purchase intention (4)	−0.116	0.484	0.553	**0.740**

Notes: The main diagonal value (in bold) is the square root of the average variance extracted.

### 4.2 Structural model and hypothesis testing

Using the structural equation model to analyze research data presented that the research model achieved an acceptable fit to the actual data: Chi-square/df = 2.750 < 3; CFI = 0.947; TLI = 0.936; IFI = 0.947 were all greater than 0.9; RMSEA = 0.063 < 0.08. The estimation result was presented in Table 4.

**Table 4:** SEM analysis result.

Hypotheses	Relationships	Beta.Std	Critical ration	p-value	Accept or reject
H1	ETH → GCI	−0.146	2.517	0.012	Accepted
H2	GCI → PCI	0.574	8.922	<0.001	Accepted
H3	ETH → PCI	−0.031	−0.619	0.536	**Rejected**
H4	GCI → INT	0.282	4.223	<0.001	Accepted
H5	PCI → INT	0.402	6.095	<0.001	Accepted
H6	ETH → INT	−0.179	−3.661	<0.001	Accepted

Notes: ETH: Consumer ethnocentrism, GCI: General country image, PCI: Product country image, INT: Intention to purchase.

**Fig. 2:**
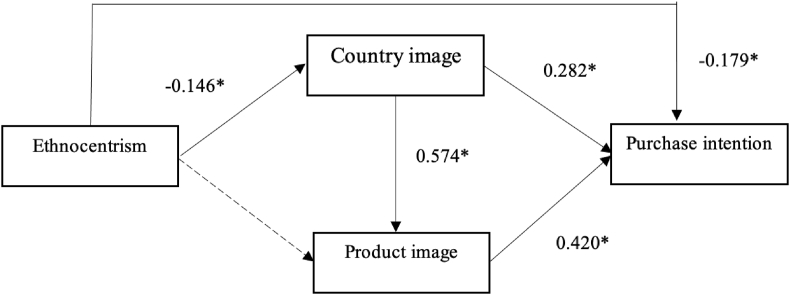
The relationship among constructs in the model. Notes: *p < 0.05.

Table 4 showed that ETH has a negative on GCI (β = −0.146 < 0, p-value = 0.012 < 0.05) and INT (β = −0.179 < 0, p-value <0.001). However, from the surveyed data, there is no evidence showing that ETH directly impacts PCI (p – value = 0.536 > 0.05). GCI has a positive impact on PCI (β = 574, p-value <0.001), and INT (β = 0.282. p-value <0.001). And PCI has a positive impact on INT (β = 0.420, p-value <0.001). On the other hands, the findings accepted hypotheses H1, H3, H4, H5, H6, and rejected hypothesis H2. The relationship among the constructs in the model could be present like Fig. 2.

## 5 Discussion and implications

This study aims to evaluate the influence of consumer ethnocentrism, a characteristic that reflects spirituality or national pride in shopping with imported products. Our study was validated by confirmatory factor analysis and structural equation modeling through model testing. As a result, most of the hypotheses posed in our model are accepted except for the direct relationship between consumer ethnocentrism and national product image (p-value >0.05). This study shows the important role of consumer ethnocentrism on the perception of imported products and consumption trends of Vietnamese consumers with products imported from China. Specifically:

The study found evidence that consumer ethnocentrism has a negative effect on the purchase intention of Chinese-origin products of Vietnamese consumers. This shows that ethnocentrism in consumption is a barrier to imported goods due to the preference of consumers to use domestic goods. This is especially emphasized with countries with an unfriendly relationship with the country the goods enter. In fact, although there is a large two-way trade relationship and the popularity of Chinese goods in Vietnam is very high, the trend of "anti-China" sentiment has always existed with Vietnamese consumers due to the politically and historically complex relationship between the two countries. The rise of China in recent decades has increased Vietnamese concerns about China’s influence in Vietnam. China’s aggressive actions such as drilling for oil and gas exploration in Vietnam’s exclusive economic zone, for example, Hai Yang Shi You 981 standoff [5], or cooperating with Cambodia, a country that shares a border with Vietnam to build a Cambodian naval base [83]. These actions further increase the anti-China sentiment among Vietnamese consumers.

This result is similar to some previous studies showing that consumer ethnocentrism has a negative effect on the intention to buy imported products [70,[84], [85], [86]]. This result also implies that domestic enterprises can make use of the nationalist mentality or consumer ethnocentrism of customers to encourage them to use alternative products/services from domestic suppliers instead of imported goods. Consumer ethnocentrism can be used as a marketing strategy to compete with imported competitors. In contrast, importers and foreign businesses need to use tactics to reduce origin identification, build international brands that have little connection with countries that have animosity to local citizens.

We also find a clear influence of country image perception and product country image on the intention to purchase imported products from China. This result is also corroborated by previous studies that show when consumers have a positive perception of a certain country's image, they also have a positive mentality toward that country's products and can promote the preference of products from that country in their purchasing decisions [48,61,62]. This also implies that importers from the domestic market or exporters from other countries can use influence strategies to change the perception of their country's image in the markets they are exporting to. In fact, this has been effectively implemented for Korean goods to Asian markets through the "Hallyu" policy of the Korean government and corporations [87,88]. Chinese enterprises can also develop similar strategies like Korean enterprises. It is necessary to create a more friendly image with local citizens, use political influences to encourage the Chinese political elite to develop more friendly foreign policies with bordering countries. Only when residents of countries under China's influence feel safe in their economic and political relations with China can anti-China sentiment be reduced. The Chinese firms need to put pressure on China's political and diplomatic circles to establish the image of China as a big but friendly and responsible in the international community.

Although consumer ethnocentrism has a negative effect on country image perception, in this study, we did not find evidence of its effect on product country image perception. This suggests that although consumer ethnocentrism has a negative effect on the perception of country image, it has little effect on the perception of the country product. This result shows that the business comes from a country with poor image perception due to historical or political issues, but its product can still be accepted if its high-quality meets consumer needs.

These research results also bring implications for businesses and policymakers with imported goods. Through the research results, we propose some suggestions for Vietnamese firms to use consumer ethnocentrism for their marketing and communication activities. Specifically, firstly, Vietnamese businesses can exploit consumer ethnocentrism through product marketing campaigns that emphasize nationalist elements in consumption such as "Vietnamese people use Vietnamese goods", "use Vietnamese goods to make Vietnamese enterprises strong”. Secondly, unified messages should be developed, emphasizing identity and national characteristics in products sold on the domestic market and using it as a transparent message to increase communication efficiency or position the business image.

## 6 Limitation

In addition to the results obtained, this study, like other studies, has some limitations. Firstly, we just focus on the group of products imported from China without comparing it with other popular imported products from countries like Japan, Korea, United States, or EU countries. To better understand whether Vietnamese consumers are generally ethnocentric or particularly toward a certain country, comparison with products of those countries that have a positive sentiment in Vietnam is also needed. Secondly, there is a minor bias in the sample of the study in terms of gender, occupation, and income variables, in which the proportion of women, students and low-income respondents is slightly higher than the rest when compared to the national data demographics. Thirdly, the research model focuses on aspects of the country's image without focusing on the image of a particular product or brand. Different types of products should be compared to see if consumer ethnocentrism is a common phenomenon or targets a certain type of product brands. Additionally, price is another factor that can affect Vietnamese consumer behavior and countries having trade agreements with Vietnam can take advantage better conditions to provide lower price products which consumers prefer. Last but not least, consumer animosity might be an important predictor that we did not include in the model. Therefore, in the future, other studies can expand the survey object with products imported from other countries add other factors like consumer animosity to the model and make more comparisons to increase the explanatory power of the model. Therefore, in the future, other studies can expand the survey object with products imported from other countries add other factors to the model and make more comparisons to increase the explanatory power of the model.

## 7 Conclusion

Our research provided some evidence about the effect of customer ethnocentrism on the perception of general country image, product country image, and purchase intention of Vietnamese customers with products imported from China. The significance of this study is to provide practical implications for both domestic commodity producers and companies importing products from China who want to successfully pursue market opportunities in Vietnam. The domestic commodity Vietnamese may use customer ethnocentrism as a competitive advantage to attract customers based on awareness of the national spirit and China's negative influence on Vietnam's economic and political relations in competing with Chinese manufacturers. In addition, the study also implies that Chinese manufacturers and importer may use some product positioning techniques that is less associated with Chinese origin to reduce the influence of customer ethnocentrism in the selection of imported products.
